# Neonatal Brain MRI: Periventricular Germinal Matrix Mimicking Hypoxic-ischemic White Matter Injuries

**DOI:** 10.1007/s00234-024-03487-9

**Published:** 2024-10-27

**Authors:** Maria Segev, Tamer Sobeh, Efrat Hadi, Chen Hoffmann, Shai Shrot

**Affiliations:** 1 Section of Neuroradiology, Division of Diagnostic Imaging, Sheba Medical Center, 2 Sheba Rd, 52621 Ramat Gan, Israel; 2 Diagnostic Ultrasound Unit, Institute of Obstetrical and Gynecological Imaging, Department of Obstetrics and Gynecology, Sheba Medical Center, Ramat-Gan, Israel; 3 School of Medicine, Faculty of Medical and Health Sciences, Tel Aviv University, Tel-Aviv, Israel

**Keywords:** Hypoxic-ischemic injury, Diffusion-weighted imaging, Development, Newborn

## Abstract

**Purpose:**

As pregnancy progresses, the germinal matrix volume decreases. Residual periventricular germinal matrix may be mistaken for hypoxic-ischemic white matter injury. This study aims to determine the prevalence and imaging characteristics of these findings.

**Methods:**

This retrospective study analyzed brain MRIs of newborns from 2012–2023, performed within the first week of life. MRIs were done for suspected hypoxic-ischemic injuries, post-natal neurological symptoms, and evaluation of prenatally diagnosed structural anomalies. Image analysis targeted the remnants of the frontal periventricular germinal matrix, assessing its imaging characteristics, including diffusion, T1, and T2 signal characteristics, and laterality. Frontal migrating cell bands were also assessed.

**Results:**

Seventy newborns were included (mean gestational age at delivery was 38.3 ± 2.1 weeks, mean scan age 5.1 ± 1.9 days). Frontal periventricular gray matter was detected in 39 newborns (90% bilateral) on T2-weighted images, negatively correlated with gestational age (r = -0.31, *p* = 0.013); none showed decreased ADC or shortened T1 signal compared with the basal ganglia. Frontal periventricular bands were found in 37 newborns (97.3% bilateral), strongly correlating with periventricular gray matter (r = 0.71, p < 0.001). No correlation was found between clinical hypoxic-ischemic injuries and these features.

**Conclusion:**

The presence of frontal periventricular gray matter observed in early neonatal MRIs, without decreased ADC values or shortened T1 signal, is developmental, reflecting a late maturation phase. Careful interpretation of MRI characteristics, including diffusion, T1, and T2 signal intensities, is necessary before attributing these findings to hypoxic-ischemic white matter injury.

**Supplementary Information:**

The online version contains supplementary material available at 10.1007/s00234-024-03487-9.

## Introduction

The germinal matrix, characterized by densely packed neuronal and glial precursors, experiences a significant reduction in volume as pregnancy progresses [1, 2]. The final phase of germinal matrix involution typically occurs around birth, specifically in regions such as the roof of the temporal horns, above the caudate nucleus, and along the lateral walls of the occipital horns of the lateral ventricles [1, 3, 4]. In T2-weighted MRI scans, the remnant germinal matrix is seen as focal periventricular hypointensities with intermediate T1 signal [5]. Concurrently, there is a dynamic migration of glial and neuronal cells during brain maturation through the deep periventricular white matter (WM). The presence of migrating cell bands is thought to indicate immature WM. Studies have shown that the prevalence of these bands decreases with increasing gestational age, and they usually disappear shortly after birth. When visible, these migrating cell bands are typically located in the deep periventricular WM of the frontal lobe, exhibiting a hypointense signal on T2-weighted MR images [1, 3].

In both preterm and term babies, MRI imaging of hypoxic-ischemic brain injury reveals different patterns. In preterm infants, hypoxic-ischemic injury often leads to white matter injury with typical features like periventricular white matter injury of prematurity [6, 7]. These appear as focal white matter lesions on MRI, either hemorrhagic or non-hemorrhagic [8], which may later result in WM volume loss over time. In term infants, hypoxic-ischemic injury predominantly affects the gray matter, with patterns of injury varying based on the maturity of the infant at birth. Nevertheless, early-term babies can also exhibit WM injury, accompanied by restricted diffusion, which is frequently observed, indicating the acuteness of these lesions[9, 10]. This difference in injury patterns between preterm and term newborns probably demonstrates a continuum of response to hypoxic-ischemic injury depending on the maturation and developmental stage of the brain at the time of the insult [10, 11].

Neonatal brain MRI studies frequently show focal periventricular elevated signal intensities on diffusion-weighted imaging near the frontal horns of the lateral ventricles, accompanied by T2 hypointensity and mild T1 hyperintensity. These findings frequently occur in isolation and do not correspond with clinical markers for hypoxic-ischemic encephalopathy (HIE). This discrepancy suggests that such imaging features might reflect normal cerebral maturation, rather than WM hypoxic-ischemic injury. The primary objective of this study is to determine the prevalence and imaging characteristics of frontal periventricular residual gray matter and to highlight the importance of careful interpretation to prevent the misdiagnosis of acute hypoxic-ischemic injuries in neonatal cerebral imaging.

## Methods

Institutional review board (IRB) approval was granted to this retrospective study, and informed consent was waived.

### Study design and population

We evaluated newborns undergoing brain MRI between 2012–2023 in our tertiary medical center. A retrospective computerized search was conducted in our institution’s Radiology Information System (RIS) (Carestream Vue RIS). The study group included newborns (≤ 7 days) referred from NICU to a brain MRI with technically adequate imaging (including T1 and T2 weighted imaging along with diffusion imaging). Clinical data was recorded from medical records, including age at the time of the study, gestational age at birth, clinical diagnosis, indication for the study, and whether the infants received hypothermia. Patients were divided into two groups: newborns undergoing the study due to suspicion of hypoxic-ischemic injury, which included cases of birth asphyxia and post-natal neurological symptoms such as seizures and apnea, and encompassing all newborns undergoing cooling; and those with no clinical suspicion of hypoxic-ischemic injury (studies performed for evaluating of suspicion of brain malformations (on prenatal or postnatal imaging), evaluating hydrocephalus, as well as suspicion of a brain hemorrhage (on prenatal or postnatal sonography). Exclusion criteria were (1) major brain malformation significantly distorting brain anatomy (as severe hydrocephalus and large vein of Galen malformation), (2) limited non-diagnostic brain imaging or technically degraded imaging.

### Imaging protocol

MRI studies were performed using either 1.5 Tesla or 3.0 Tesla systems from various manufacturers (GE Medical Systems, USA; Ingenia, Philips Medical Systems, Netherlands, or Prisma, Siemens Healthcare, Germany). All patients had routine clinical MRI scans, including T1 and T2 weighted imaging, diffusion-weighted imaging (axial 2D spin-echo sequence with EPI readout, with two b values of 0 and 1000 mm2/s) (detailed acquisition parameters appear in Table 1 on supplementary material), and heme-sensitive sequences, as T2* gradient echo (GRE) or susceptibility-weighted imaging (SWI). Time-of-flight MR angiography and post-contrast T1 imaging were added depending on specific clinical scenarios.

### Image analysis

**Fig. 1:**
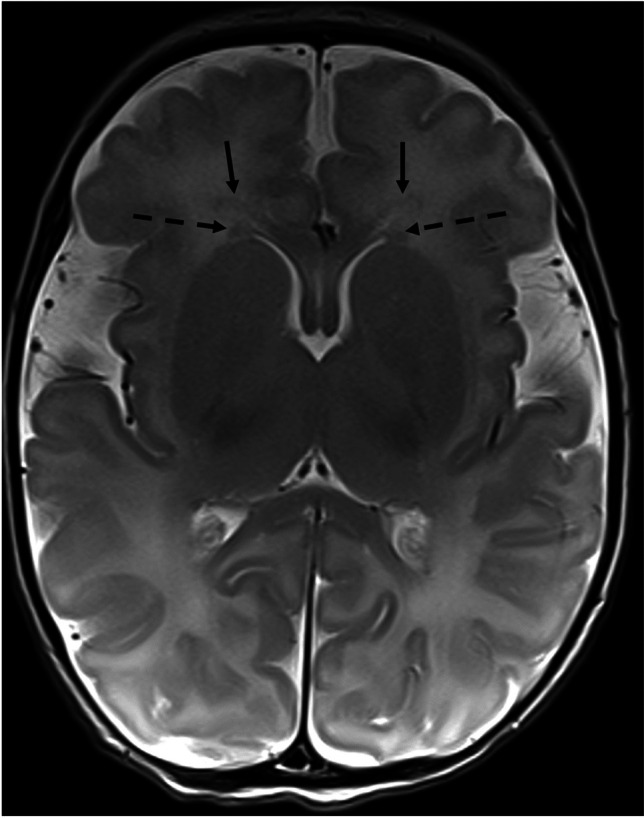
T2 weighted image showing frontal periventricular gray matter over the head of the caudate nucleus (dashed arrows) and frontal periventricular bands of migrating cells (solid arrows)

MRI images were analyzed for frontal periventricular germinal matrix over the head of the caudate nucleus (“frontal periventricular gray matter”) and frontal periventricular bands of migrating cells (“frontal periventricular bands”) (Fig. 1). Imaging characteristics included assessment of DWI and ADC signal intensity (qualitative assessment of high, iso, and low compared to the adjacent opercular cortex on DWI and corresponding ADC maps), T1 (high, iso, and low compared to opercular cortex), and T2 signal characteristics (compared with adjacent white matter), and laterality. Signal abnormalities in heme-sensitive sequences (GRE or SWI) were also recorded. Additional imaging findings, including hemorrhage, ischemia, infarctions, or other structural anomalies or malformations, were recorded from formal radiology reports. Two board-certified radiologists independently scored all images (M.S., a fellow in pediatric radiology with five years of experience in radiology, and S.S., a senior pediatric neuroradiologist with 7 years of experience in pediatric neuroradiology). Discrepancies were settled by the senior pediatric neuroradiologist (S.S.).

### Statistical analysis

Categoric variables were expressed as numbers and percentages. Continuous variables were expressed as mean and standard deviation. Intraclass correlation was used to assess inter-observer variability. Spearman’s were performed to analyze the correlations. A 2-tailed p < 0.05 was considered statistically significant. Analyses were performed with SPSS (Version 29.0, 2023; IBM, Armonk, New York).

## Results

**Fig. 2:**
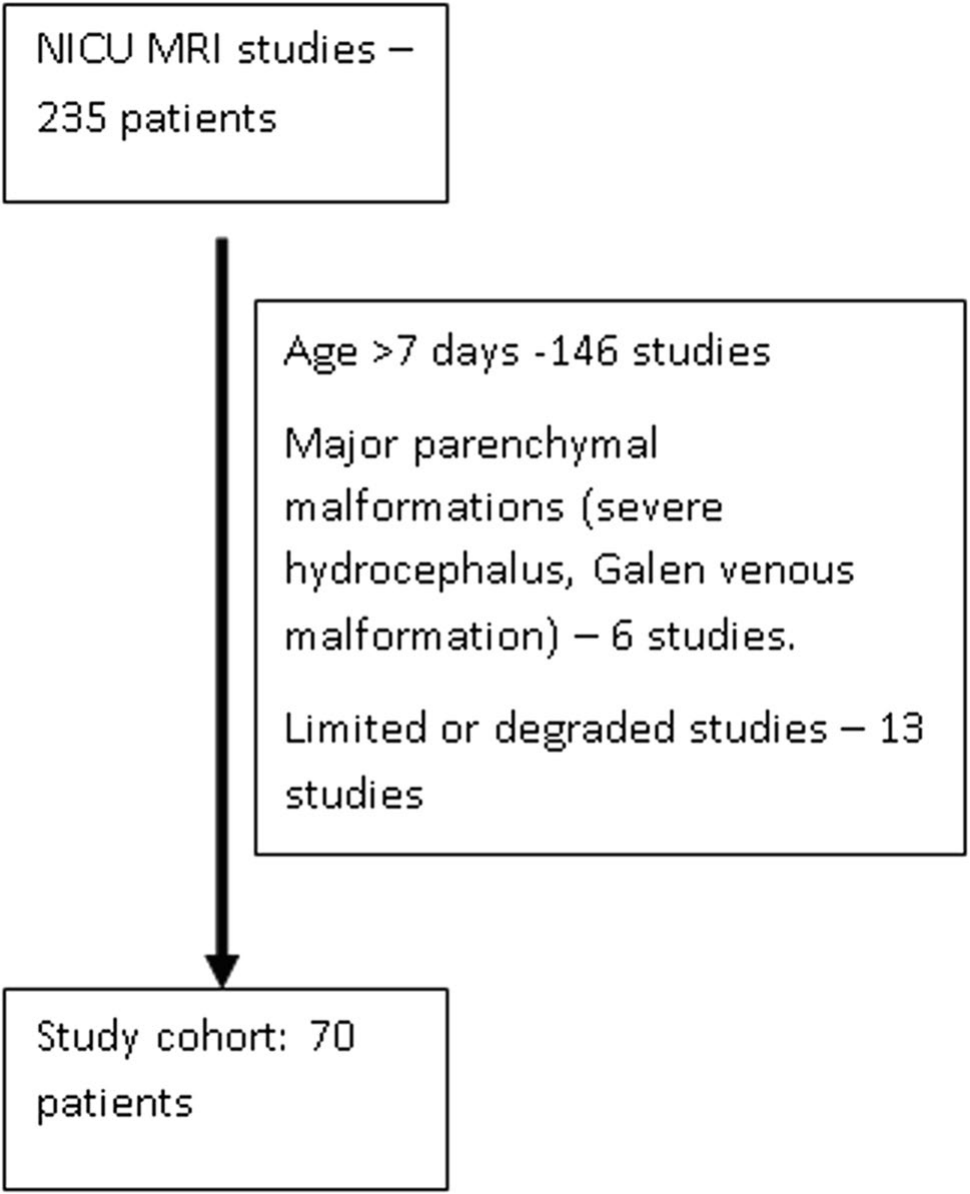
Study flowchart

*Study cohort and characteristics*—235 newborns had a brain MRI while hospitalized in the NICU. Of these, 70 patients met the inclusion criteria (Fig. 2 and Table 1). The mean age at MR scan was 5.1 days (SD 1.9 days), and the mean age at delivery was 38.3 weeks of gestation (SD 2.1 weeks). 51.4% (*n* = 36) were males. 82 percent of patients were term (≥ 37 weeks). In 3 patients, this GA was not available.

**Table 1:** Demographic and clinical characteristics of the study cohort

Age at MRI Scan (mean and SD^*^)	5.1 days (SD^*^ 1.9)
Gestational Age at Birth (mean SD^*^)	38.3 weeks (SD^*^ 2.1)
Preterm Births (< 37 weeks)	12/67 (18%)
Gender (Male:Female)	36:34
Indication for the Study	
- Hypoxic-Ischemic and Resuscitation-Related Conditions	19 (27.1%)
- Neurologic Symptoms	24 (34.3%)
- Developmental and Structural Anomalies	21 (28.6%)
- Hydrocephalus	4 (5.7%)
- Brain Hemorrhage	3 (4.3%)
Hypothermia	25/70 (35.7%)
Magnet Strength (1.5T:3.0T)	4:66

^*^SD- Standard deviation

*Indications for MRI—*MR studies were initiated for 19 patients based on suspicion of hypoxic-ischemic injury, evidenced by either the necessity for postnatal resuscitation or the occurrence of birth asphyxia. Additionally, neurological symptoms and conditions, such as seizures, abnormal neurological examination findings, hypotonia, or apnea, led to the MR in 23 patients. 25 newborns were treated with a cooling protocol. For 21 patients, the investigation was prompted by potential developmental or structural anomalies inferred from prenatal imaging or initial head sonography studies. The diagnosis of hydrocephalus via ultrasound imaging was the investigative rationale in four patients. Overall, considering clinical indication or application of the cooling protocol, 47 out of 70 patients underwent MRI due to suspected hypoxic-ischemic injury.

**Fig. 3:**
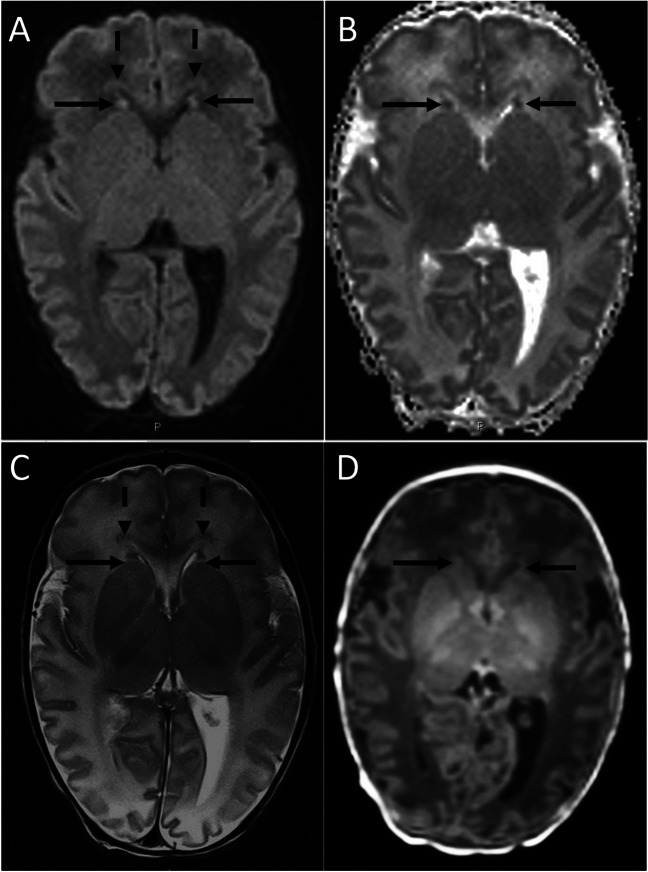
MRI of a newborn scanned at 7 days for suspected seizure activity (35 weeks GA) demonstrating frontal periventricular gray matter (arrows) and migrating bands (dashed arrows) on (**A**) DWI, corresponding ADC map (**B**) and T2-Weighted images (**C**). The DWI signal is higher compared to the nearby white matter, but it is similar to the opercular cortex. Note T1 signal is similar to the cortex (**D**)

*Imaging findings (*Table 2*)—*Frontal periventricular gray matter abnormalities were evident in 39 newborns as focal hypointensities on T2-weighted images (Fig. 3), with 90% of these cases being bilateral (*n* = 35). Frontal periventricular gray matter abnormalities were observed as a focal high signal on DWI in 21 newborns. None of the patients displayed decreased signal on ADC maps when compared to the opercular cortex or basal ganglia at the same MR level. On T1-weighted images, frontal periventricular gray matter was identified in 25 newborns, all of which were isointense to the cortex\basal ganglia.

**Table 2:** Imaging characteristics of Frontal periventricular gray matter and periventricular bands

	T2^a^	Bilaterality	T1^a,b^	DWI^a^	ADC^a,b^
Frontal periventricular gray matter	39/70	35/39	25/70 (lower-0, iso-25, higher-0)	21/70	36 (4-lower, 32-iso, 0-higher)
Frontal periventricular bands	37/70	36/37	11/70 (5-hypo, 6-iso, 0-hyper)	6/70	31/70 (0-lower, 25-iso, 6-higher)

^a^visibility of frontal periventricular gray matter and periventricular bands

^b^T1 and ADC characteristics compared with adjacent cortex or basal ganglia appear in parentheses

Frontal periventricular bands were evident in 37 newborns as focal hypointensities on T2-weighted images, with 97.3% of these cases being bilateral (*n* = 36). Frontal periventricular bands were observed as a focal high signal on DWI in 6 newborns. All these lesions did not show a decreased signal on ADC maps compared to the adjacent opercular cortex, with 35.7% being isointense and 8.6% showing a higher signal on ADC maps compared to the adjacent opercular cortex. On T1-weighted images, frontal periventricular bands were identified in 11 newborns, with 5 exhibiting hypointense signals and six being isointense compared with adjacent opercular cortex. None of the frontal periventricular gray matter and periventricular bands showed low signal on heme-sensitive sequences.

There was a negative correlation between frontal periventricular gray matter and GA of the neonates seen on T2-weighted and DW images (*r* = -0.31, *p* = 0.013, and *r* = -0.27, *p* = 0.03, respectively). The presence of these periventricular bands was associated with younger age at the exam (*r* = -027, *r* = 0.023). There was a strong correlation between frontal periventricular gray matter and periventricular bands (*r* = 0.71, p < 0.001). There was no correlation between frontal periventricular gray matter or periventricular bands and clinical suspicion for hypoxic-ischemic injury (*p* = 0.7 for periventricular gray matter and *p* = 0.08 for periventricular bands).

Interobserver variability – For frontal periventricular gray matter and frontal periventricular bands, ICC reached a good agreement on T2 WI (ICC = 0.71). Lower ICC was found on DWI images ( ICC = 0.36).

**Fig. 4:**
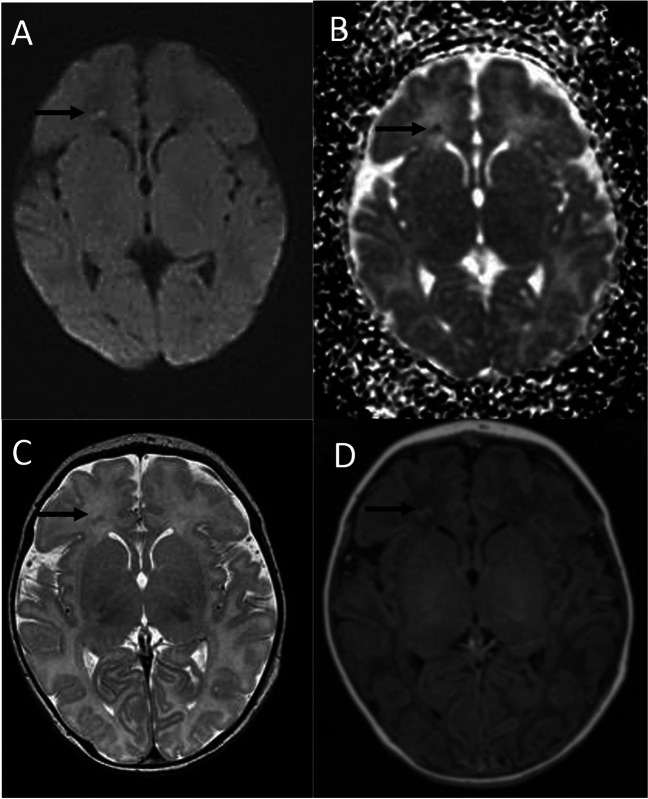
MRI of 6 days newborn performed brain MRI for suspected seizure activity (39 weeks GA) demonstrating frontal periventricular white matter focal injury characterized with restricted diffusion (arrow on **A**) with corresponding low ADC values (arrow on **B**). Note decreased T2 signal and high T1 signal (arrows on **C** and **D**)

Additional imaging findings – Significant additional imaging findings were brain hemorrhages (*n* = 9), focal hypoxic-ischemic white-matter lesions (*n* = 20, Fig. 4), and structural anomalies and malformations (*n* = 11). 30 MR scans were reported as normal.

## Discussion

In over fifty percent of initial magnetic resonance (MR) examinations conducted on newborns, frontal periventricular gray matter was observed in T2-weighted and diffusion-weighted imaging (DWI) modalities, frequently associated with frontal periventricular bands. These MR studies were performed for a range of clinical reasons, of which a third was not related to suspected hypoxic-ischemic injury. Typically, the imaging revealed that the frontal periventricular gray matter appeared symmetric. Furthermore, an inverse relationship was identified with gestational age, indicating that these developmental structures become less conspicuous as brain development progresses.

As MRI usage in preterm and term infants grows, an increase in the detection of subtle punctate lesions in white matter is observed [12]. Recent studies have linked the increased risk of white matter injury in preterm infants to the sensitivity of specific brain cells, like late oligodendrocytes. Research indicates infants with focal WM lesions show lower fractional anisotropy, suggesting widespread microstructural changes not visible on conventional MRI [13]. While WM injury is common in preterm infants, it is also seen in term newborns, particularly those with congenital heart disease and neonatal encephalopathy [9, 10, 14]. These findings highlight the significance of comprehending the diverse manifestations and underlying mechanisms of focal white matter lesions across various newborn populations.

While hemorrhagic WM lesions can easily be identified on SWI, non-hemorrhagic WM lesions often show restricted diffusion (i.e., increased signal intensity on DWI with decreased ADC values), attributed to possible inflammatory or ischemic etiology. Restricted diffusion appears only on early scans, i.e., during the first week after injury [15]. In our study, apparently restricted diffusion of the frontal periventricular gray matter with lower ADC signal relative to adjacent non-myelinated WM can be misdiagnosed as focal WM injury, though ADC values are not low compared with adjacent opercular cortex. Furthermore, non-hemorrhagic WM lesions observed in early MRI scans commonly show a pronounced increased T1 signal, likely due to the onset of early gliosis [16, 17]. In our cohort, the similarity in the T1 signal of the frontal periventricular gray matter to the cortex or basal ganglia further makes the diagnosis of focal WM lesions unlikely. In our study, most patients' focal frontal periventricular gray matter appears symmetric, which is less characteristic of hypoxic-ischemic WM injury. The assessment of frontal periventricular regions in newborns using multiple MRI sequences is critical to distinguish between periventricular maturation remnants and focal white matter hypoxic-ischemic injury. The utilization of T2-weighted, DWI, ADC maps, and T1-weighted and SWI sequences provided valuable insights into the nature of these focal abnormalities.

Similar to periventricular bands, frontal periventricular gray matter is most probably developmental structures involuting brain maturation and represents residual fetal structures. Starting from the end of the second trimester of pregnancy, there is a significant diminution in the volume of the germinal matrix, consisting of densely packed neuronal and glial progenitors [1, 3, 4]. As gestation approaches term, the germinal matrix contracts to a minimal cellular layer adjacent to the ventricles. This final phase of involution of the germinal matrix is observed in near-term gestation overlying the caudate nucleus head as well in other sites as the apex of the temporal horns of the lateral ventricles and along the lateral wall of the occipital horn of the lateral ventricles. These relatively compact cells might result in a signal similar to the cortex, I.e., shorter T1 and T2 and reduced diffusivity, compared with surrounding non-myelinated WM. These embryological remnants are probably common in the early postnatal period, especially in preterms and young-term newborns. Our findings align with previously established data regarding the higher frequency of periventricular bands in younger premature infants scanned at term [5], extending these developmental imaging findings also to term newborns imaged shortly after birth.

Our study faced several limitations, including its retrospective design, leading to a heterogeneous cohort with MRI scans performed at various ages for different clinical reasons. This diversity and the absence of neurodevelopmental outcome assessments limited our ability to draw broader conclusions. However, we included newborns without clinical suspicion of hypoxic-ischemic injury and those who were evaluated for structural abnormalities detected in prenatal imaging with no clinical suspicion of hypoxic-ischemic injury as an “internal non-hypoxic-ischemic control group”. This internal control group enables highlighting the common findings of residual germinal matrix mimicking hypoxic-ischemic injury. Another limitation stems from the retrospective design of the study, which involved the use of different 3T scanners and varying sequence parameters. This introduced scanner-related variability in the detection of periventricular gray matter and periventricular bands.

To conclude, our study reveals that early MRIs of newborns frequently show frontal periventricular gray matter, often alongside frontal periventricular bands, suggesting developmental rather than hypoxic-ischemic origins. These findings, seen in preterm and term infants, highlight the complexity of diagnosing focal white matter (WM) injuries, emphasizing the need to carefully interpret MRI characteristics like diffusion and signal intensity.

## Supplementary Information

Below is the link to the electronic supplementary material.

Supplementary file1 (DOCX 15 KB)

## Data Availability

The datasets generated and analyzed during the current study are available from the corresponding author upon reasonable request.
