# Supplementary material for: Neonatal Brain MRI: Periventricular Germinal Matrix Mimicking Hypoxic-ischemic White Matter Injuries
Source: Neuroradiology. 2024 Oct 27;67(2):499–505. doi: 10.1007/s00234-024-03487-9 (PMC11893625; doi:10.1007/s00234-024-03487-9)
Supplement: Supplementary file 1 — Supplementary file1 (DOCX 15 KB) [file 234_2024_3487_MOESM1_ESM.docx]

**Table 1 -** Acquisition parameters of newborn brain MRI, performed in various scanners.

|  | **GE scanner** | | | | | | | **Philips scanner** | | | **Simense scanner** | | | |
| --- | --- | --- | --- | --- | --- | --- | --- | --- | --- | --- | --- | --- | --- | --- |
| **Coil** | Head coil (16-channel) | | | | | | | Head coil (32-channel coil) | | | Head coil (64-channel) | | | |
|  | T1 | | | | | T2 | DWI | T1 | T2 | DWI | T1 | | T2 | DWI |
| **Slice thickness** | 3 | | | | | 3 | 3 | 1 mm | 2-4 | 3 | 1 mm | | 3mm | 3mm |
| **Gap** | 0.3 | | | | 0.3 | | 0.3 | no | 0.4 | 0.3 | no | | 0.3 | 0.3 |
| **Field-of-view (FOV)** | 23-24 cm | | | | | | | 23-24 cm | | | 24-25 cm | | | |
| **Matrix size** | 256/256 | | | 256/256 | | | 256/256 | 448/448 | 320/320 | 224/224 | 256/256 | | 384/384 | 192/192 |
| **Echo time (TE) (ms)** | 12-15 | | 120-130 | | | | 100-105 | 7 | 100-120 | 70 | 900 | 100-105 | | 55-60 |
| **Repetition time (TR) (ms)** | 790 | 7450-7500 | | | | | 7450-7500 | 3 | 8800-9300 | 5200 | 1800 | 2950-3000 | | 6250 |
